# Representation in participatory healthcare decision‐making

**DOI:** 10.1111/hex.13521

**Published:** 2022-05-08

**Authors:** Emma L. Moore, Lakshmi Chandrasekaran, Holly Sheldon‐Wilson, Janneke Scholtz

**Affiliations:** ^1^ Population Health Research Institute St George's University of London London UK; ^2^ Undergraduate Medical School Southampton University Medical School Southampton UK; ^3^ Psychiatry Department St Pancras Hospital London UK

In a fascinating paper, Holetzek and Holmberg emphasize the importance of representation when conducting patient and public involvement (PPI) in research.^1^ We therefore reviewed our recent PPI work and considered whether our lay participants met their criteria in terms of responsiveness, characteristics and skills.

PPI allows the incorporation of the unique perspectives that participants bring to research, which are crucial in the design of many studies.^2^ In December 2021, we visited a London technical/further education college to explore the opinions of female students on the proposed patient literature for a feasibility study of using vaginal lactoferrin (a prebiotic protein derived from milk) instead of antibiotics/antifungals to treat bacterial vaginosis (BV) and thrush.^3^, ^4^ Our previous online survey of 82 women (mean age 22) found that 84% would be happy to try vaginal lactoferrin as a natural alternative to oral treatment for vaginal discharge. However, our funders were keen for us to also explore the views of ethnically diverse teenage women, who are often underrepresented in research.

Fifteen female students, mean age of 18 years (range: 16–28) joined our PPI group. Seven (47%) identified as ‘Black/African/Caribbean/Black British’, five (33%) as ‘White’, two as ‘Asian/Asian British’ and one as ‘Mixed/multiple ethnic groups’. Twelve participants (80%) had heard of BV or thrush, but only one had ever been tested for these conditions.

Most participants stated that the patient information was ‘clear’ and ‘easy to understand’. However, a few found it ‘repetitive’ or ‘too long’ with ‘a lot of words.’ Concerns were expressed about the storage of their personal details and the lack of information on possible side effects of lactoferrin. Despite this, nine participants (60%) said they would be interested in joining the study. One commented: ‘My mum uses yogurt for thrush and has done for years. I think everyone should use it’.

To what extent did our PPI group participants meet Holetzek and Holmberg's^1^ criteria for representation?

1. Responsiveness—No. Our lay participants were probably unaware of the views of nonparticipants, particularly as none had personal experience of BV/thrush.
2. Characteristics—Possibly. Nearly half our participants were of black ethnicity, a group in whom BV is more prevalent.
3. Skills—Possibly. Some members of the group appeared to show ‘interest and motivation’,^1^ but it was unclear if they could encourage group consensus.

Holetzek and Holmberg^1^ point out that as in our study, the representation will always be imperfect. It is clearly important that researchers are aware of this. In addition, we may not have applied the representation criteria as intended. However, as a result of our PPI work, we have improved the patient information sheet by adding more information on data protection and on possible side effects of lactoferrin. We have also designed a concise, user‐friendly study flier.

## PATIENT OR PUBLIC CONTRIBUTION

This letter contributes to improving public participation in healthcare research.

## DATA AVAILABILITY STATEMENT

Data are available on request.
